# Genome-wide transcriptome profiling of radish (*Raphanus sativus* L.) in response to vernalization

**DOI:** 10.1371/journal.pone.0177594

**Published:** 2017-05-12

**Authors:** Chen Liu, Shufen Wang, Wenling Xu, Xianxian Liu

**Affiliations:** Institute of Vegetables and Flowers, Shandong Academy of Agricultural Sciences, Shandong Key Laboratory of Greenhouse Vegetable Biology, Shandong Branch of National Vegetable Improvement Center, Jinan, Shandong, People's Republic of China; Chungnam National University, REPUBLIC OF KOREA

## Abstract

Vernalization is a key process for premature bolting. Although many studies on vernalization have been reported, the molecular mechanism of vernalization is still largely unknown in radish. In this study, we sequenced the transcriptomes of radish seedlings at three different time points during vernalization. More than 36 million clean reads were generated for each sample and the portions mapped to the reference genome were all above 67.0%. Our results show that the differentially expressed genes (DEGs) between room temperature and the early stage of vernalization (4,845) are the most in all treatments pairs. A series of vernalization related genes, including two *FLOWERING LOCUS C* (*FLC*) genes, were screened according to the annotations. A total of 775 genes were also filtered as the vernalization related candidates based on their expression profiles. Cold stress responsive genes were also analyzed to further confirm the sequencing result. Several key genes in vernalization or cold stress response were validated by quantitative RT-PCR (RT-qPCR). This study identified a number of genes that may be involved in vernalization, which are useful for other functional genomics research in radish.

## Introduction

Radish (*Raphanus sativus* L.) is an economically important root vegetable crop grown worldwide, particularly in China, Japan, Korea, and Southeast Asia [1]. For the nutrient-rich tuberous root, many breeding efforts on radish have been devoted to developing varieties with different size [2–4], color [5–9], cultivation season [5, 10, 11] and other characteristics [12–14]. Although all-season radish is available in many areas now, there are still a lot of practical problems in production, among which premature bolting is one of the most prominent. Before bolting, the plant needs a period of low temperature to accomplish vernalization. A better understanding of the molecular mechanism of vernalization will be helpful to solve these practical problems such as premature bolting.

In the past several decades, many efforts were made to illustrate the molecular mechanisms of vernalization in various plants, among which *Arabidopsis thaliana* has been studied extensively. In *Arabidopsis thaliana*, the vernalization requirement is mainly due to the expression level of the *FLC* gene [15]. Low temperature can reduce the level of the DNA methylation and affect the *FLC* expression [16]. FLC is a MADS-box transcription factor and functions as a flowering suppressor during vegetative growth, by directly binding to the floral integrators downstream, such as *FLOWERING LOCUS T* (*FT*), *FLOWERINGLOCUS D* (*FD*) and *SUPPRESSOR OF OVEREXPRESSIONOF CONSTANS 1* (*SOC1*) [17–19]. Additionally, in the upstream of *FLC*, the vernalization pathway includes other genes, such as *VERNALIZATION1* (*VRN1*), *VERNALIZATION2* (*VRN2*), *VERNALIZATION INSENSITIVE 3* (*VIN3*) and *VIN3-LIKE1* (*VIL1*)/*VERNALIZATION5* (*VRN5*) [20]. Study on a *vin3* mutant showed that the expression level of *FLC* was not repressed even when the plant underwent a long period of low temperature [21]. In the plants with the *vrn1* and *vrn2* mutants, though cold stress can reduce the *FLC* expression, the repression is reversed when the temperature rises again [22, 23]. These results indicate that *VIN3* participates in the suppression of *FLC* at the beginning of vernalization, while *VRN1* and *VRN2* function to maintain the low level of *FLC* expression. In addition to *Arabidopsis*, other crops with different gene regulatory circuitries of vernalization have also been investigated in recent years [24–28]. For radish, several genes related to vernalization have been found [29–31]. However, the molecular mechanism of vernalization is still largely unknown.

The emergence of the next generation sequencing (NGS) technology has improved the throughput and shortened the cycle time of sequencing, which facilitates molecular studies at the transcriptome and genome levels. The application of the NGS technology to transcriptome analysis, namely RNA-Seq, offers an efficient and inexpensive way for transcriptome studies. Transcriptome sequencing on radish has led to the discovery of many critical genes related to certain characteristics, and the development of numerous molecular markers [32–34]. However, the majority of the transcriptome sequencing analyses were analyzed by *de novo* assembly because the reference genome was unavailable when these studies were constructed. The recently released genome sequence [35, 36] provides us a new strategy for radish transcriptome sequencing with better coverage and accuracy.

In this study, we analyzed the radish transcriptome during vernalization with RNA-Seq, using the radish genome as a reference [36]. The vernalization-related genes and the gene expression patterns were studied by three treatments with different cold exposure schemes. Our study provides an important opportunity to advance our understanding of the molecular mechanism of vernalization in radish and other plants.

## Results

### Illumina sequencing and mapping against radish reference genomes

For each of the three treatments, three replicates were prepared and each replicate included ten individuals. The samples were labeled as RT1, RT2, and RT3 for the room temperature treatment (RT), VE1, VE2, and VE3 for the early stage of vernalization (VE), and VL1, VL2, and VL3 for the late stage of vernalization (VL), respectively. A total of nine cDNA libraries were constructed and sequenced by paired-end sequencing on an Illumina HiSeq 2500 platform. More than 36 million raw reads were generated for each library, and the portions of clean reads were all above 99.60% (Table 1). Two reported radish genomes, “RSA_r1.0” [35] and “rsgv1” [36], were used for mapping analysis. For RSA_r1.0, the clean reads for VE1, VE2, VE3, VL1, VL2, VL3, RT1, RT2 and RT3 were 66.93%, 67.28%, 67.02%, 66.69%, 65.34%, 66.70%, 67.42%, 67.88% and 68.01%, respectively. The portions of clean reads were higher when rsgv1 was used as the reference genome, which ranged from 67.85% to 69.91% (Table 1). Hence, the rsgv1 genome was used as the reference for subsequent analysis. For the reference genome, more than 67% of the clean reads in each library were uniquely mapped, while less than 1.5% was mapped to multiple positions. A total of 558 new genes were identified after filtering out the sequences that contain only one exon or encode a peptide less than 50 amino acids (S1 Table).

**Table 1:** Summary of Illumina transcriptome sequencing from radish seedlings.

Type	Raw reads	Clean reads		Mapped Reads^a^	
	Total number	Total number	Percent of raw reads	Total number	Percent of clean reads
VE1	51,257,924	51,140,846	99.77%	35,309,896	69.04%
VE2	45,305,236	45,187,684	99.74%	30,661,120	67.85%
VE3	41,966,002	41,846,074	99.71%	28,729,949	68.66%
VL1	41,286,054	41,179,192	99.74%	28,418,114	69.01%
VL2	37,777,842	37,643,682	99.64%	25,769,729	68.46%
VL3	36,974,214	36,858,198	99.69%	25,330,052	68.72%
RT1	40,208,132	40,099,820	99.73%	27,841,148	69.43%
RT2	47,534,910	47,408,220	99.73%	32,604,257	68.77%
RT3	45,867,076	45,741,058	99.73%	31,978,882	69.91%

^a^the mapped reads of the reference genome rsgvl.

FPKM: the fragments per kilobase of transcript per million fragments mapped; RT: room temperature treatment; VE: the early stage of vernalization; VL: the late stage of vernalization.

### Identification of differentially expressed genes during vernalization

**Fig 1:**
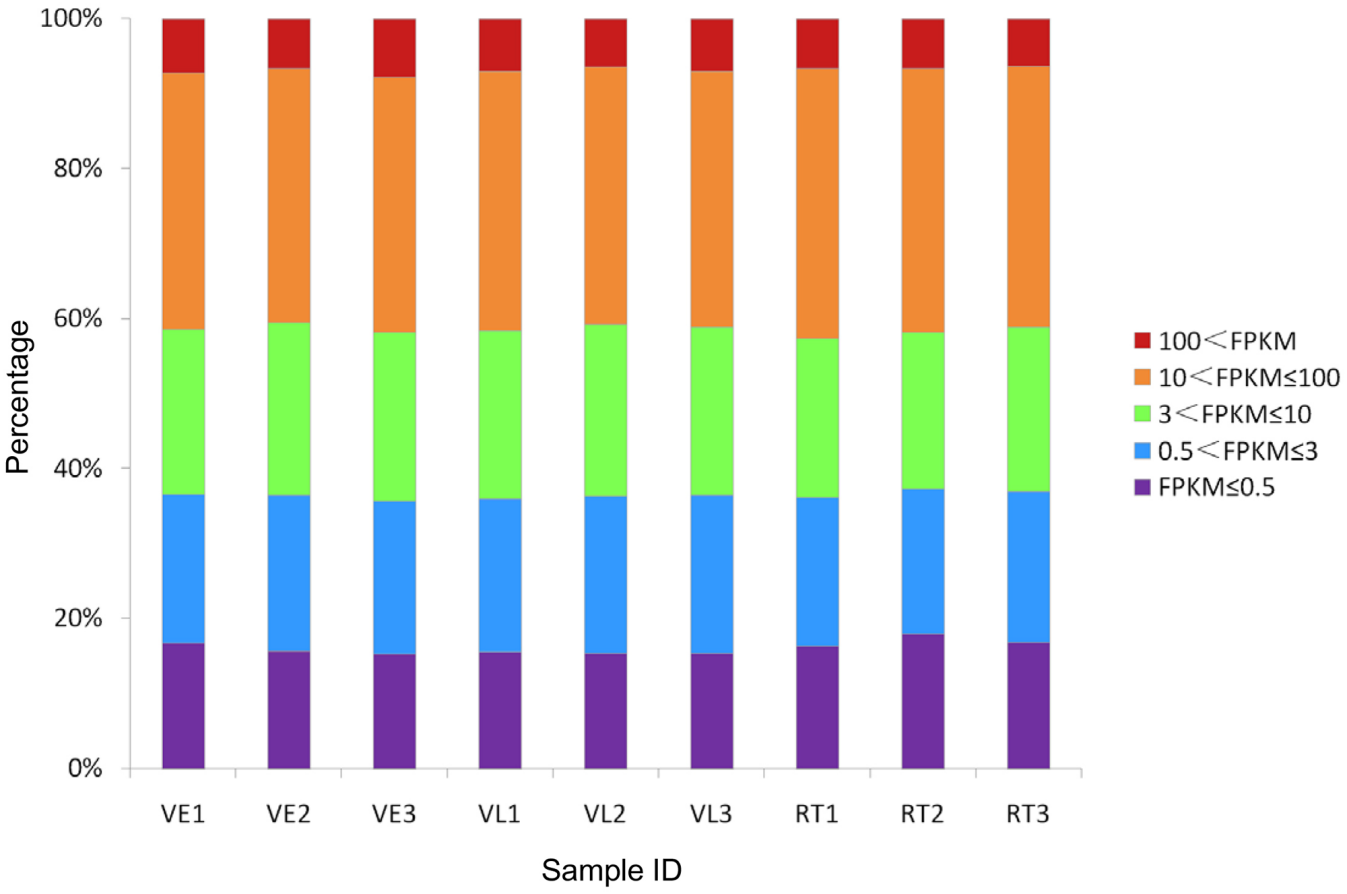
Global analysis of transcriptome datasets of the nine samples. The y-axis indicates the percentage of expressed transcripts after filtering.

**Fig 2:**
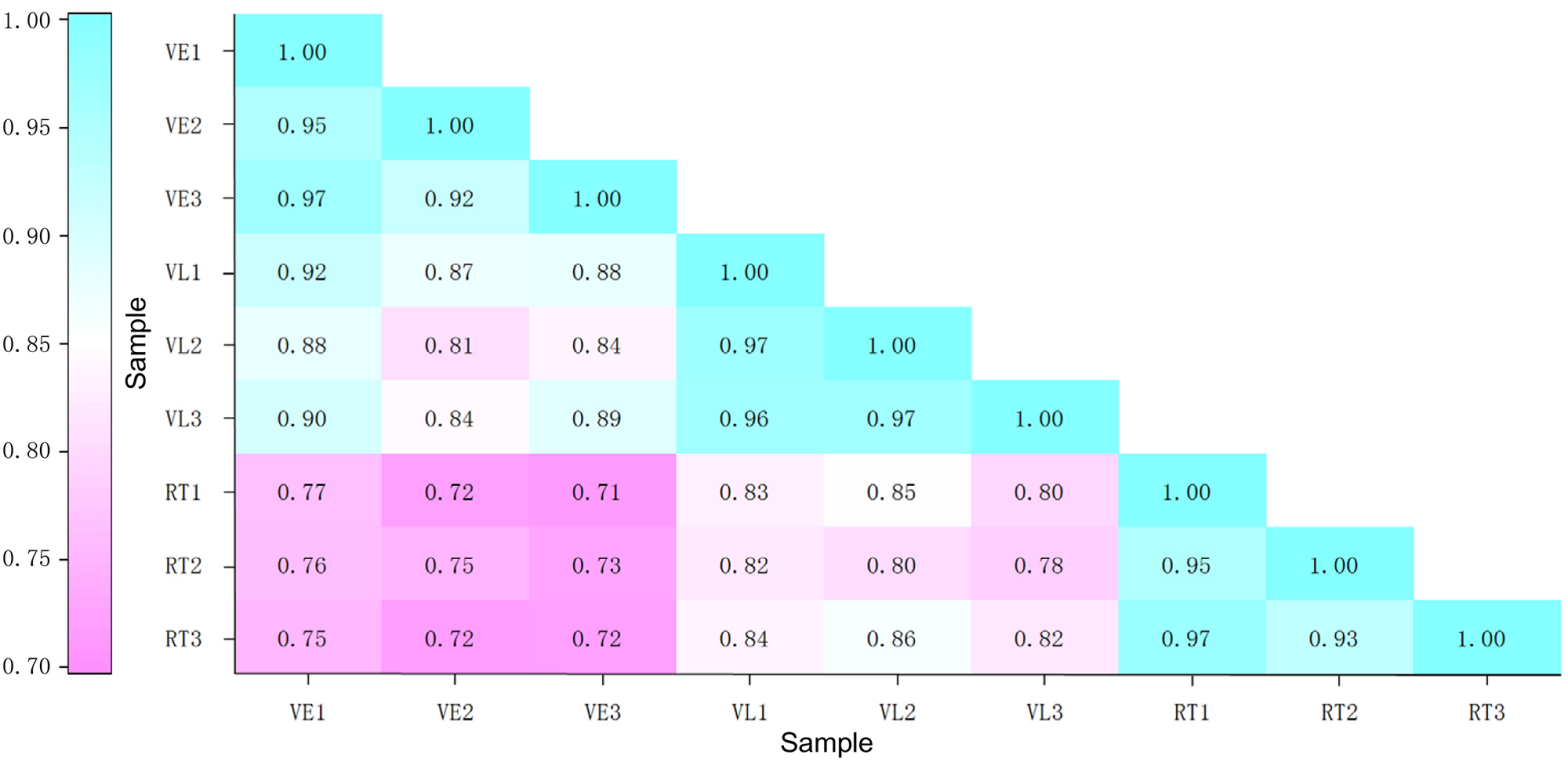
The correlation coefficients (*R*^*2*^) among the samples.

The transcripts were classified into five categories according to the fragments per kilobase of transcript per million fragments mapped (FPKM) [37] (Fig 1). The genes with FPKM between 10 and 100 belonged to the largest group, followed those with FPKM between 3 and 10. According to the FPKM value, the correlations of the gene expression of the nine samples, especially the three biological replicates, were assessed, and the correlation coefficients (*R*^*2*^) [38] were all above 0.91 among the replicates (Fig 2). Genes expressed in different treatments were screened. A total of 29,535, 29,416 and 29,540 genes were expressed in all three replicates in VE, VL and RT, respectively (S2 Table). More than 92% (27,304) were expressed in all three treatments, while only a small portion (40, 35 and 129 for VE, VL and RT, respectively) was expressed specifically in one treatment.

**Fig 3:**
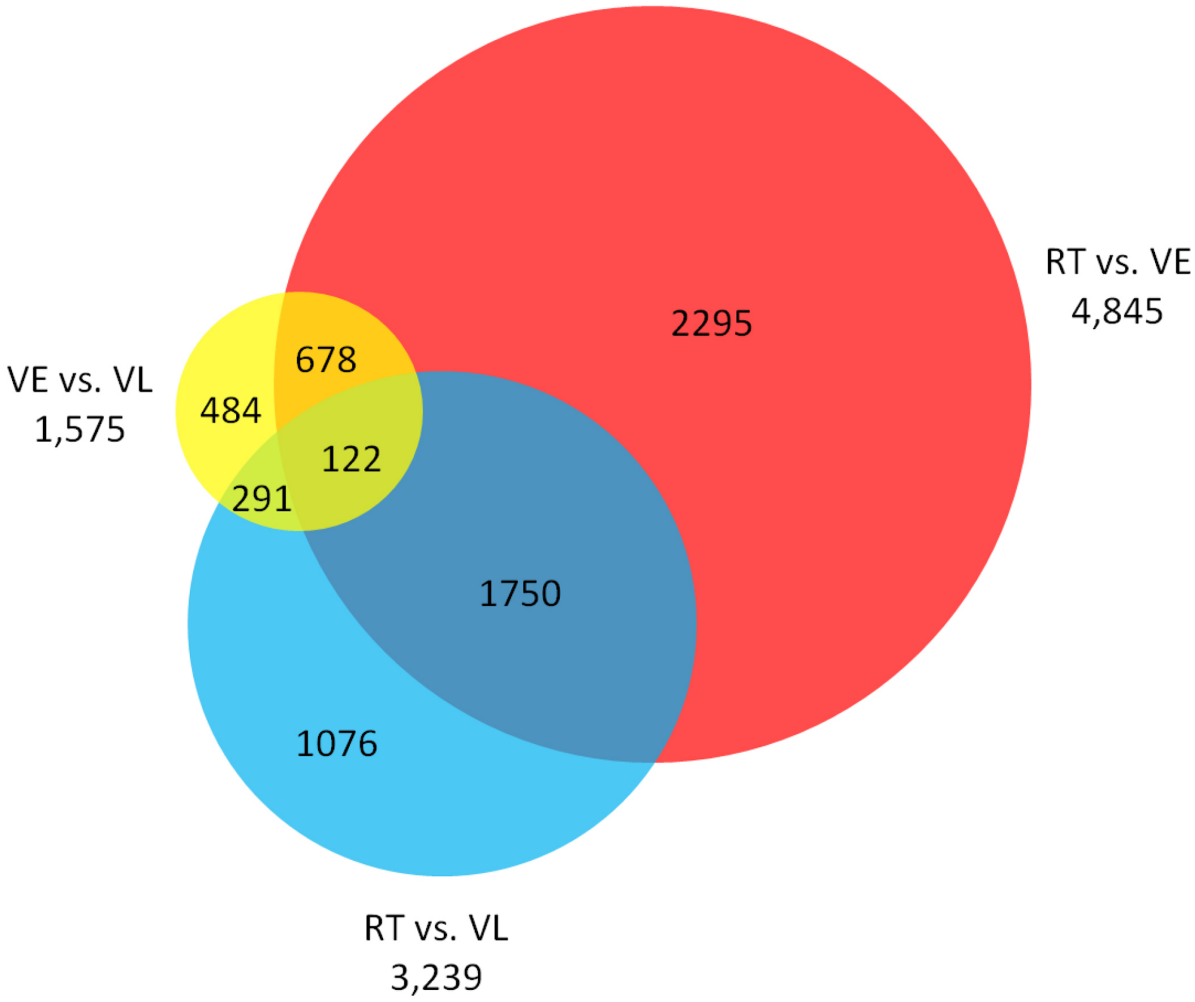
Venn diagram of numbers of DEGs between different treatments.

DEGs was identified by the DEGseq package [39], and a total of 1,575 (706 up- and 869 down-regulated), 4,845 (2,675 up- and 2,170 down-regulated), and 3,239 (1,653 up- and 1,586 down-regulated) genes were differentially expressed in the treatment pairs, VE vs. VL, RT vs.VE, and RT vs. VL, respectively. All DEGs were divided into seven groups according to the expression profiles, among which the expression of 122 genes was significant different in all three treatment pairs, and the genes only differentially expressed between RT and VE belonged to the largest group (Fig 3).

### Gene Ontology (GO) enrichment of DEGs during vernalization

GO enrichment of the DEGs was conducted using the GO database. A total of 1,419, 4,363 and 2,925 DEGs from the three corresponding treatment pairs were annotated, respectively. All the GO terms were ranked based on the Kolmogorov-Smirnov (KS) value [40], and only the top three GO terms of the three main groups (cellular component, molecular function and biological process) of each treatment pair were picked for comparison (S3 Table). In the biological process category, “pyrimidine ribonucleotide biosynthetic process” was shared between RT vs. VE and VE vs. VL, while “single-organism process” and “single-organism cellular process” were shared between RT vs. VL and VE vs. VL. “Protein binding” and “ATP binding” from the molecular function category were found in all treatment pairs. The term “sequence-specific DNA binding transcription factor activity” was shared between RT vs. VL and VE vs. VL. No term in the cellular component category was shared.

### Metabolic pathway analysis of DEGs

Kyoto Encyclopedia of Genes and Genomes (KEGG) enrichment analysis of DEGs, based on the KEGG database, was performed to identify significantly altered pathways involved in vernalization. In total, 377, 1,309 and 773 DEGs from the VE vs. VL, RT vs.VE, and RT vs. VL treatment pairs were annotated, respectively. Only 3 pathways were significantly enriched with Q value ≤ 0.01. For VE vs. VL, “ribosome biogenesis in eukaryotes” and “purine metabolism” were enriched. “Ribosome” and “ribosome biogenesis in eukaryotes” were enriched in RT vs.VE. No pathway was significantly enriched in RT vs. VL. In addition, pathways with the most DEGs in both RT vs. VE and RT vs. VL were “Ribosome”, followed by “Biosynthesis of amino acids” and “Carbon metabolism”. The corresponding pathways in VE vs. VL were “Ribosome biogenesis in eukaryotes”, “Protein processing in endoplasmic reticulum”, “Purine metabolism” and “Ribosome” (S1–S3 Figs).

### Screen vernalization related candidates according to the expression profile

**Fig 4:**
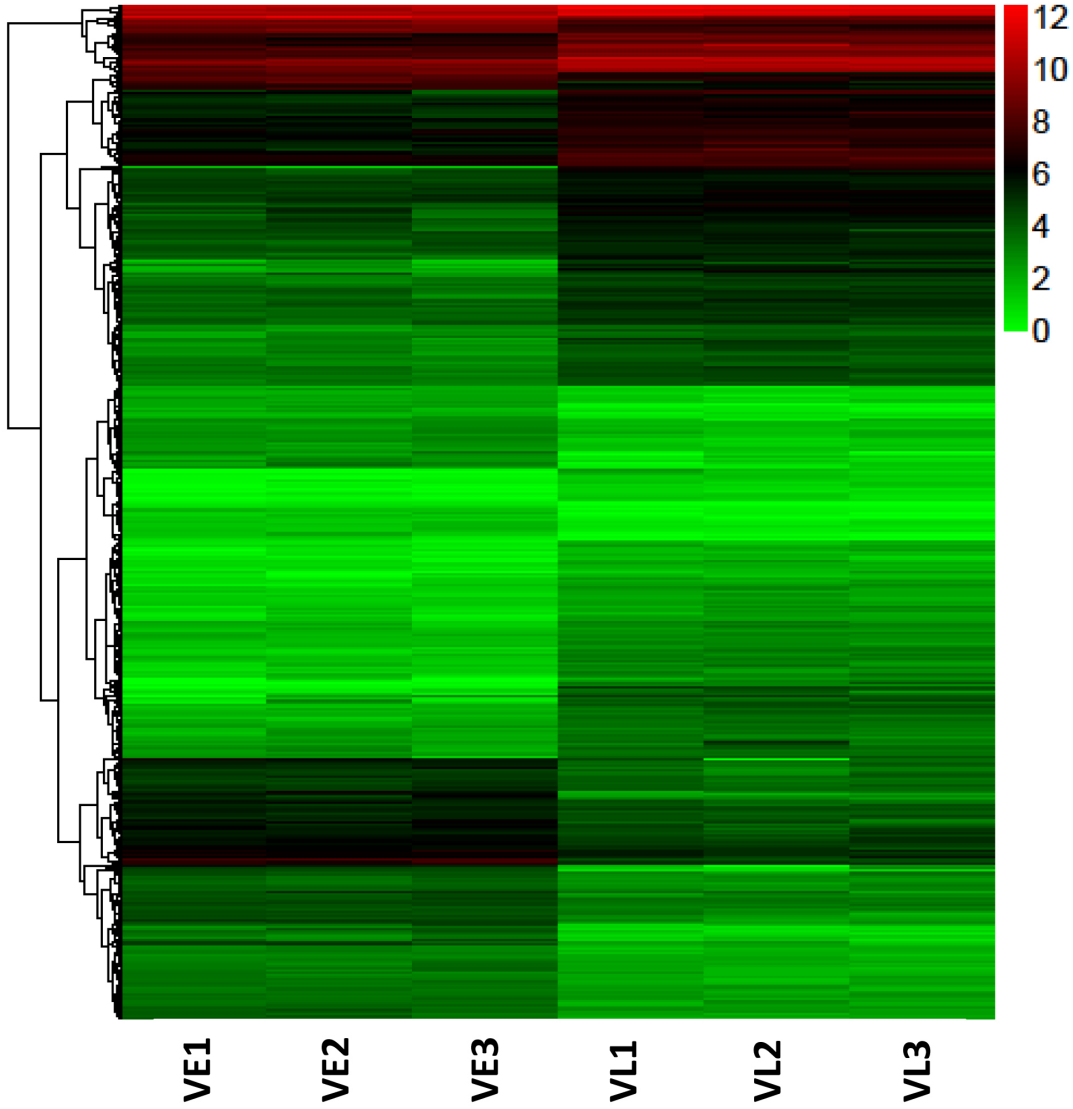
Hierarchical clustering graph of genes with expression difference in VE vs. VL but not in RT vs. VE.

Vernalization is a mechanism for plants to avoid flowering in an improper season. Plants have to undergo a period of cold, which is long enough to ensure the winter has passed. Hence, genes with expression changes that only occur after a long period of cold are more likely to function in the regulation of vernalization. In this study, these genes belong to two groups of the DEGs, which show expression difference in VE vs. VL and not in RT vs. VE (Fig 3). A total of 775 (459 up- and 316 down-regulated) vernalization related candidate genes were screened (Fig 4). The screened genes accounted for a small portion (11.57%) of the DEGs and the up-regulated candidates were more than the down-regulated. Except for infinite and infinitesimal log2Ratio values, the maximum absolute values of log2Ratio for up- and down-regulated genes were 8.95 and 8.91 in all DEGs, while the corresponding values were 7.55 and 5.62 in the screened candidates.

### Validation of DEGs by quantitative RT-PCR

**Fig 5:**
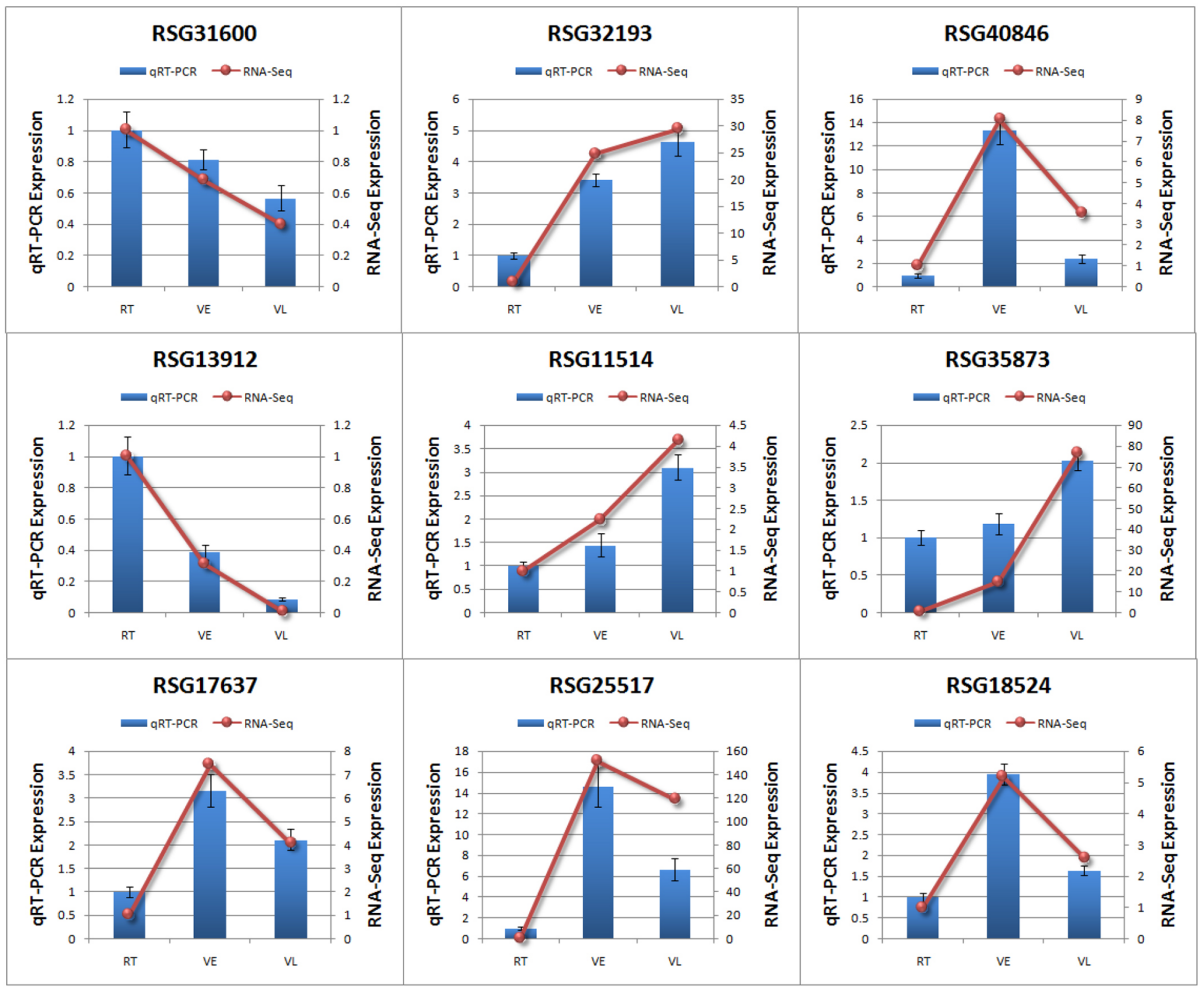
qRT-PCR verification of differentially expressed genes. Gene expression differences analyzed by qRT-PCR and RNA-seq are both exhibited. For qRT-PCR, error bars indicate the standard deviation (STDEV) for the replicates in each experiment.

To verify the DEGs, five and four genes related to vernalization [15] and cold stress response [41] were selected for quantitative RT-PCR, respectively. The result showed that all genes exhibited the same expression tendency as in the RNA-Seq result, indicating that our RNA-Seq results are reliable (Fig 5).

## Discussion

The molecular mechanism of vernalization is still largely unknown in radish. One high throughput sequencing analysis aiming at radish vernalization has been reported, which was *de novo* assembled and mainly focused on the *FLC* genes [31]. In this study, we analyzed the radish transcriptome before and after vernalization with a reference genome and identified a series of vernalization-related genes. Our experiment includes three treatments in which, the plants were exposed to low temperature for 0, 3 and 20 days, respectively. A total of 39,118 transcripts were detected. Among the three treatments, the experiment for the pair RT vs.VE discovered more DEGs than the other two experiments, with the least number of DEGs in the experiment for VE vs. VL. The large amount of DEGs in RT vs.VE may be partly because of the cold stress responsive genes which had expression changes immediately after exposure to low temperature.

**Fig 6:**
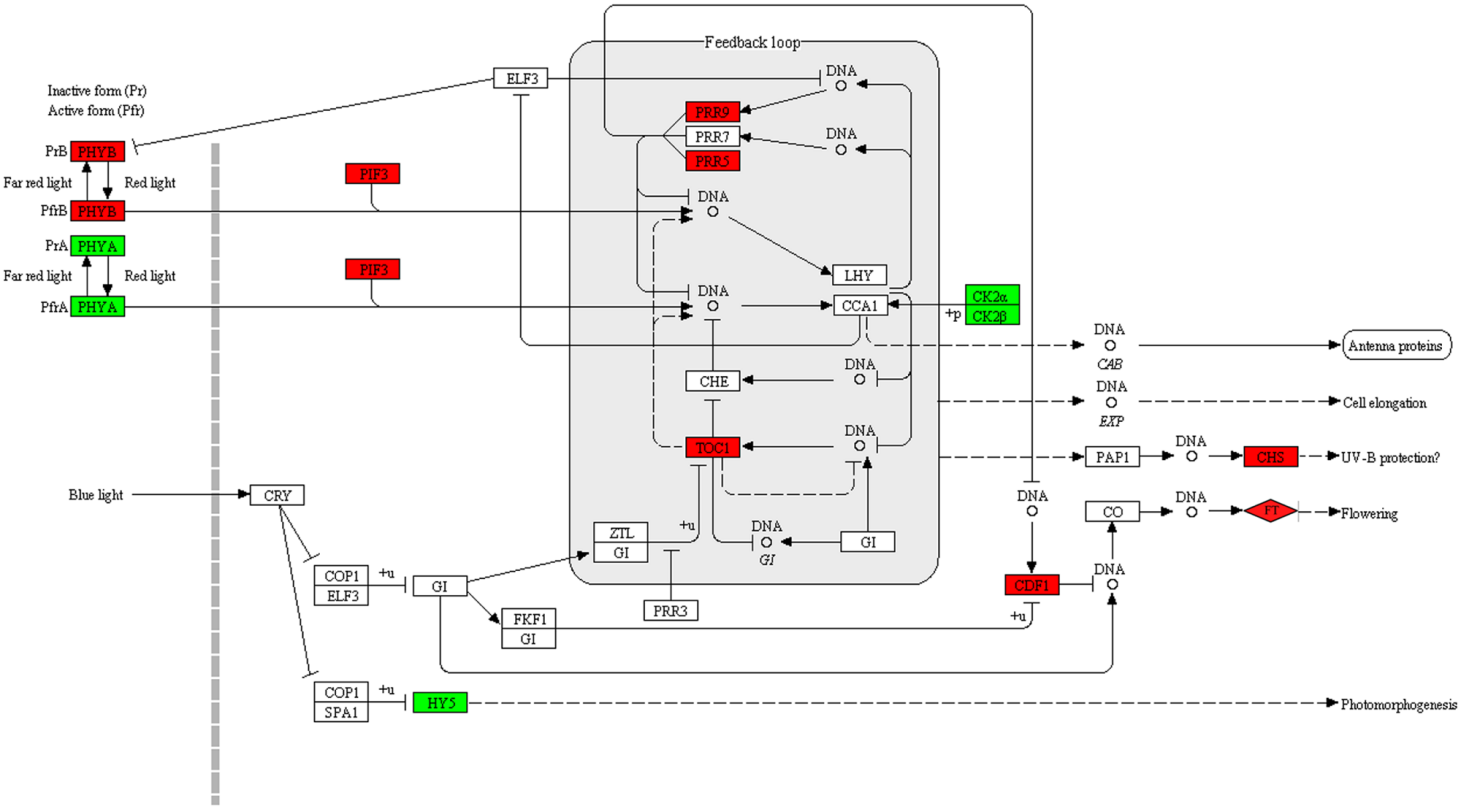
The pathway of circadian rhythm in radish. The genes in colored rectangular box are DEGs in RT vs. VE, and that in colored rhombic box are DEGs in VE vs. VL. The up-regulated genes are in red and the down-regulated genes are in green.

Vernalization is one of the four major flowering regulation pathways in plants. Before flowering, a period of low temperature is needed for winter plant to accomplish vernalization, during which a series of related genes are regulated. To date, *Arabidopsis* is the model plant used for the investigation of the vernalization mechanism. According to the regulatory network of *Arabidopsis* [15], several key genes, including *FLC*, *VRN1*, *VRN2*, *VIN3*, *VIL1*/*VRN5*, *FT*, *FD* and *SOC1*, were analyzed in our result. Three *FLC* genes were reported in a previous study [31], but only two were annotated in the reference genome. Two *FLC* genes (RSG13912 and RSG31600) were detected in our study, and both were down-regulated (S4 Table). RSG13912 was down-regulated in the whole process and RSG31600 was down-regulated only in RT vs. VL. For the upstream of *FLC*, 2, 2, 10 and 6 genes were found in the genome annotated as *VIN3*, *VRN1*, *VRN2* and *VIL1*/*VRN5*, respectively, while the numbers of *VRN2* (5) and *VIL1*/*VRN5* (5) in the present study were less than the genome (S4 Table). In *Arabidopsis*, *VRN1*, *VRN2* and *VIL1*/*VRN5* are constitutively expressed regardless of vernalization. Our result showed that the expressions of most genes did not change noticeably during vernalization, except 1 *VIL1*/*VRN5*. The *VIN3* gene was reported to be up-regulated in the process of vernalization in previous studies [21, 42], which was also identified in this study. The two *VIN3* genes did not have the same expression pattern; RSG35873 was up-regulated in the whole process, and RSG11514 was up-regulated only in RT vs. VL. *FT*, *FD* and *SOC1* are at the downstream of *FLC*, which contains 1, 3 and 3 members in the radish transcriptome (S4 Table). Only the member of *FT* showed an increase of the expression. This gene involves in the flowering process of the “circadian rhythm-plant” pathway (ko04712). The up-regulation in the late stage of vernalization indicates its important role in promoting flowering (Fig 6).

In addition to the key genes in vernalization, the members of previously reported protein complexes that regulate the expression of *FLC* [15] were also studied in our result. The complexes involved in the activation of *FLC* in *Arabidopsis* include the COMPASS complex [43], the RAD6-BRE1 complex [44], the PAF1 complex [45], the FRI complex [46] and the SWR1 complex [46]. Among the FLC activators, the expression of most components of the complexes did not change during vernalization. Only 1, 1, 2 and 2 genes in COMPASS, PAF1, FRI and RAD6-BRE1 were down-regulated, respectively (S5 Table). Regarding the repressors of FLC, there are mainly 2 complexes, PRC2 and PRC1 [47, 48]. Similar to the FLC activators, the expression of most components of PRC2 and PRC1 did not change during vernalization (S5 Table). Three and one genes in PRC2 and PRC1 were up-regulated, respectively. These results indicate that the majority of the complex members are constitutively expressed.

Among the seven groups of the DEGs, two groups that showed expression difference in VE vs. VL and not in RT vs. VE were selected as vernalization related candidates. The up-regulated genes were more than the down-regulated genes, and the fold change of the up-regulated genes was larger than the down-regulated genes, except the infinite and infinitesimal values. Among the up-regulated genes with the most dramatic changes (log2Ratio ≥ 4), the majority encode proteins that are involved in plant growth and development, such as glycine-rich protein, beta-1, 3-glucanase, cytochrome P450, etc (S6 Table). The thioglucoside glucohydrolase gene (*TGG1*) [49] was also identified, implying it may play an important role in vernalization in addition to defenses against insects and disease. In the dramatically down-regulated genes, one gene encodes indole-3-acetic acid (IAA) inducible 32 was filtered. Moreover, the “plant hormone signal transduction” pathway (ko04705) also showed a down-regulation of *AUX*/*IAA* (S4 Fig). Previous studies showed that decrease of IAA can promote the process of flower bud differentiation [50, 51]. The down-regulated *IAA32* gene in this study indicates that IAA may also play an important role in the process of vernalization.

To further verify our sequencing result, the COR genes and CBF genes which constitute the predominant cold signaling pathway in plant [41] were selected for expression analysis. Among the 13 genes annotated as COR, 12 CORs were identified in this study (S7 Table). Among the COR genes, 5 were differentially expressed in vernalization, and up-regulated since the early stage of vernalization and did not change between VE and VL. All 8 CBF genes were detected, among which, only 2 were up-regulated in RT vs. VE and down-regulated in VE vs. VL. The increases in the expression of the CORs and CBFs after cold exposure are consistent with the reports in other plants [52–55].

## Materials and methods

### Plant material and RNA extraction

A green radish inbred line “2^#^” was used for this study. The seeds were obtained from the Institute of Vegetables and Flowers, Shandong Academy of Agricultural Sciences. Three cold exposure treatments were constructed with different exposure time (0, 3 and 20 days). These treatments stand for room temperature, early stage of vernalization and late stage of vernalization respectively. For room temperature treatment, the seeds germinated and grew in culture dishes with wet filter paper at room temperature. The seeds for the early stage of vernalization treatment also germinated and grew in culture dishes at room temperature and then the culture dishes were placed in the refrigerator at 4°C for 3 days, while the germinating seeds for the late stage of vernalization were transferred to 4°C for 20 days. All seedlings were cultivated in the dark and sampled at the same time when they were of the same size and stored at -80°C until RNA extraction.

Total RNA was isolated using Trizol reagent (Invitrogen, USA) following the standard protocol. The concentration of the total RNA was determined by NanoDrop (Thermo Scientific, USA), and the RNA integrity value (RIN) was checked using RNA 6000 Pico LabChip of Agilent 2100 Bioanalyzer (Agilent, USA).

### cDNA library construction and sequencing

Enrichment of mRNA was conducted with oligo magnetic adsorption. The enriched mRNA was randomly fragmented by the fragmentation buffer. Severed as a template, mRNA was used for the first-strand cDNA synthesis with random hexamers. The second-strand cDNA was synthesized using DNA polymerase I and purified using AMPure XP beads (Agencourt, Beverly, MA, USA). Sequencing adaptors were linked to the purified cDNA, and the cDNA fragments of a suitable length were then selected by AMPure XP beads. Finally, nine double-strand Illumina libraries were obtained by PCR amplification. The libraries were sequenced by the Illumina HiSeq 2500 system (Biomarker Technologies Co., Ltd, Beijing, China). All datasets from the Illumina sequencing platform can be found in the Short Read Archive (SRA) database of the National Center for Biotechnology Information (NCBI) under accession number SRP093947.

### Data processing

Raw data were processed to remove primers and adaptor sequences. Low quality reads were filtered out and the reads with more than 80% portion Q ≥ 30 were obtained as clean reads. Clean reads were aligned with the TopHat2 software [56] using two reported radish genomes [35, 36] as references. Reads aligned to the genome sequence were named as mapped reads, and only mapped reads were selected for subsequent analysis. The number of the mapped reads was used for the calculation of alignment efficiency and for the selection of the optimal reference genome. The mapped reads were assembled by the Cufflinks software [57], and compared with the reference genome to uncover new genes.

### Analysis of differentially expressed genes

The gene expression level was calculated using the FPKM method [37]. Correlations of the biological replicates were evaluated by calculating the Pearson’s Correlation Coefficient [38]. Differentially expressed genes between different treatments were identified by the DEGseq package [39]. The significant differences in the expression levels were assessed using false discovery rate (FDR) and log2Ratio with a threshold “FDR < 0.01 and the absolute value of log2Ratio ≥ 1”. To annotate the biological functions of the DEGs, GO enrichment analysis was performed using the GO database (http://geneontology.org/), and KEGG enrichment analysis was performed using the KEGG database (http://www.genome.jp/kegg/).

### Quantitative RT-PCR validation

To validate the sequencing result and the DEGs, qRT-PCR was conducted. The first strand cDNA synthesis and qRT-PCR analysis were conducted using the TransScript One-Step gDNA Removal and cDNA Synthesis SuperMix (AT311, TransGen Biotech, Beijing, China) and the TransStart Tip Green qPCR SuperMix (AQ141, TransGen Biotech, Beijing, China), respectively. The *ACTIN* gene was chosen as a constitutive expression control in the qRT-PCR analysis. The gene-specific primers of the validated DEGs and *ACTIN* are listed in S8 Table. PCR reactions were performed on an IQ5 Real-Time PCR System (BIO-RAD, Hercules, CA, USA) with the following cycling parameters: 95°C for 2 min, followed by 45 cycles at 95°C for 15 s, and 60°C for 70 s. All reactions were performed with three replicates. Gene expression levels were calculated using the delta—delta Ct method [58].

## Conclusions

We presented a comprehensive analysis of the gene expression profiles in radish during vernalization, using the latest published genome sequence as the reference. A series of vernalization related genes were identified according to the annotations and the expression patterns. Cold stress responsive genes were also analyzed to further confirm the sequencing result. This study offers important insights into the molecular mechanism of vernalization in radish.

## Supporting information

S1 Fig KEGG annotation of the DEGs in VE vs. VL. (JPG)

S2 Fig KEGG annotation of the DEGs in RT vs. VE. (JPG)

S3 Fig KEGG annotation of the DEGs in RT vs. VL. (JPG)

S4 Fig The pathway of plant hormone signal transduction in VE vs. VL. (JPG)

S1 Table Information of the new genes. (XLS)

S2 Table Overview of the mapping result. (XLS)

S3 Table Top three GO terms of the three main groups ranked by the KS value. (XLS)

S4 Table Candidates of the key genes in vernalization from radish transcriptome. (XLS)

S5 Table Identification of members of the complexes that regulate the expression of FLC. (XLS)

S6 Table Genes that showed expression difference in VE vs. VL and not in RT vs. VE with |log2Ratio| ≥ 4. (XLS)

S7 Table Identification of the key genes in response to cold stress. (XLS)

S8 Table Primers used for qRT-PCR verification of differentially expressed genes. (XLS)

## References

[1] Lu Z, Liu L, Li X, Gong Y, Hou X, Zhu X, et al Analysis and evaluation of nutritional quality in Chinese radish (*Raphanus sativus* L.). Agricultural Sciences in China. 2008;7(7):823–30.

[2] Dhankhar BS, Kishore N, Dhankhar SK. Hisar Selection-1: A new variety of radish. Haryana journal of horticultural science. 2006;35(3/4):324–5.

[3] Zhang B, Zhao L. A new spring radish hybrid ‘Lingcui’. Acta Horticulturae Sinica. 2012;39(2):399–400.

[4] Han T, Xu L, Yang X, Tan J, Song Y, Chen X, et al A new early spring white radish F1 hybrid—‘Weiluobo No.4’. China Vegetables. 2013(20):93–5.

[5] Liu X, Xu W, Liu C, Zhao W, Wang S. Breeding of a new early spring radish hybrid Tianzhengluobo 14 with tolerance to bolting. Shandong Agricultural Sciences. 2016;48(8):23–5.

[6] Kalia P, Chandel KS, Pathania NK. Palam Hriday: a new radish. Indian Horticulture. 2004;49(1):33.

[7] Zhang H, Wang X, Zhang A, Jia R, Zhang X. A new spring radish hybrid, Chunhongyou. Journal of Changjiang Vegetables. 2011;11:50–1.

[8] Han T, Yang X, Xu L, Tan J, Song Y. A new plate leaves and red flesh cultivar 'WeiLuobo2'. Acta Horticulturae Sinica. 2009;36(1):151.

[9] Wang S, Liu X, Wang W, Xu W. A New Radish F1 Hybrid—'Tianzhengluobo No. 10'. Shandong Agricultural Sciences. 2012;44(9):133–4.

[10] Zhang H, Wang X, Zhang A. Breeding of Xiahong No.2, a new summer and autumn radish cultivar. Journal of Changjiang Vegetables. 2014(22):13–4.

[11] Wang S, Liu X, Liu C, Li Q, Zhang Z, Zhao Z, et al Breeding of a new radish hybrid Tianzhengluobo No. 13. Shandong Agricultural Sciences. 2016;48(7):32–4.

[12] Lee YP, Park S, Lim C, Kim H, Lim H, Ahn Y, et al Discovery of a novel cytoplasmic male-sterility and its restorer lines in radish (*Raphanus sativus* L.). Theor Appl Genet. 2008;117(6):905–13. 10.1007/s00122-008-0830-3 18597066

[13] Nieuwhof M. Breeding for low nitrate content in radish (*Raphanus sativus* L.). Euphytica. 1991;55:171–7.

[14] Park S, Lee SS, Yoon MK, Mok ILG, Park HG. Development of uniform F1 hybrid varieties of Korean radish using self-incompatibility in double-crossing. International Journal of Plant Breeding. 2007;1(2):119–22.

[15] Kim DH, Sung S. Genetic and epigenetic mechanisms underlying vernalization. The Arabidopsis book. 2014;12:e0171 10.1199/tab.0171 24653667 PMC3952384

[16] Kim DH, Doyle MR, Sung S, Amasino RM. Vernalization: winter and the timing of flowering in plants. Annual Review of Cell and Developmental Biology. 2009;25:277–99. 10.1146/annurev.cellbio.042308.113411 19575660

[17] Helliwell CA, Wood CC, Robertson M, James Peacock W, Dennis ES. The Arabidopsis FLC protein interacts directly in vivo with SOC1 and FT chromatin and is part of a high-molecular-weight protein complex. the Plant Joural. 2006;46(2):183–92. 10.1111/j.1365-313X.2006.02686.x 16623882

[18] Xu F, Rong X, Huang X, Cheng S. Recent advances of *flowering locus T* gene in higher plants. International Journal of Molecular Sciences. 2012;13(3):3773–81. 10.3390/ijms13033773 22489182 PMC3317742

[19] Searle I, He Y, Turck F, Vincent C, Fornara F, Krober S, et al The transcription factor FLC confers a flowering response to vernalization by repressing meristem competence and systemic signaling in Arabidopsis. Genes & Development. 2006;20(7):898–912. 16600915 10.1101/gad.373506 PMC1472290

[20] He Y, Amasino RM. Role of chromatin modification in flowering-time control. Trends in Plant science. 2005;10(1):30–5. 10.1016/j.tplants.2004.11.003 15642521

[21] Levy YY, Mesnage S, Mylne JS, Gendall AR, Dean C. Multiple roles of *Arabidopsis VRN1* in vernalization and flowering time control. Science. 2002;297(5579):243–6. 10.1126/science.1072147 12114624

[22] Sung S, Amasino RM. Vernalization in *Arabidopsis thaliana* is mediated by the PHD finger protein VIN3. Nature. 2004;427(6970):159–64. 10.1038/nature02195 14712276

[23] Gendall AR, Levy YY, Wilson A, Dean C. The *VERNALIZATION 2* gene mediates the epigenetic regulation of vernalization in *Arabidopsis*. Cell. 2001;107(4):525–35. 11719192 10.1016/s0092-8674(01)00573-6

[24] Zou X, Suppanz I, Raman H, Hou J, Wang J, Long Y, et al Comparative analysis of FLC homologues in Brassicaceae provides insight into their role in the evolution of oilseed rape. PloS One. 2012;7(9):e45751 10.1371/journal.pone.0045751 23029223 PMC3459951

[25] Bergonzi S, Albani MC, Ver Loren van Themaat E, Nordstrom KJ, Wang R, Schneeberger K, et al Mechanisms of age-dependent response to winter temperature in perennial flowering of *Arabis alpina*. Science. 2013;340(6136):1094–7. 10.1126/science.1234116 23723236

[26] Oliver SN, Finnegan EJ, Dennis ES, Peacock WJ, Trevaskis B. Vernalization-induced flowering in cereals is associated with changes in histone methylation at the *VERNALIZATION1* gene. Proc Natl Acad Sci USA. 2009;106(20):8386–91. 10.1073/pnas.0903566106 19416817 PMC2677093

[27] Pin PA, Zhang W, Vogt SH, Dally N, Buttner B, Schulze-Buxloh G, et al The role of a pseudo-response regulator gene in life cycle adaptation and domestication of beet. Current Biology. 2012;22(12):1095–101. 10.1016/j.cub.2012.04.007 22608508

[28] Sun M, Qi X, Hou L, Xu X, Zhu Z, Li M. Gene Expression Analysis of *Pak Choi* in Response to Vernalization. PloS One. 2015;10(10):e0141446 10.1371/journal.pone.0141446 26517271 PMC4627790

[29] Guo J, Zu Y, Wu Y, Zheng J, Mei Y. Prediction and analysis of flowering related genes RFLCs in *Raphanus* species. Acta Agriculturae Zhejiangensis. 2014;26(3):656–60.

[30] Park HJ, Jung WY, Lee SS, Lee Jw, Kim Y-S, Cho HS. Physiological and molecular characterization of two inbred radish lines with different bolting times. Journal of Plant Biotechnology. 2015;42(3):215–22.

[31] Yi G, Park H, Kim J-S, Chae WB, Park S, Huh JH. Identification of three *FLOWERING LOCUS C* genes responsible for vernalization response in radish (*Raphanus sativus* L.). Horticulture, Environment, and Biotechnology. 2015;55(6):548–56.

[32] Mei S, Liu T, Wang Z. Comparative transcriptome profile of the cytoplasmic male sterile and fertile floral buds of radish (*Raphanus sativus* L.). International Journal of Molecular Sciences. 2016;17(1). 10.3390/ijms17010042 PMC4730287 26751440

[33] Wang S, Wang X, He Q, Liu X, Xu W, Li L, et al Transcriptome analysis of the roots at early and late seedling stages using Illumina paired-end sequencing and development of EST-SSR markers in radish. Plant Cell Reports. 2012;31(8):1437–47. 10.1007/s00299-012-1259-3 22476438

[34] Wang Y, Pan Y, Liu Z, Zhu X, Zhai L, Xu L, et al De novo transcriptome sequencing of radish (*Raphanus sativus* L.) and analysis of major genes involved in glucosinolate metabolism. BMC genomics. 2013;14:836 10.1186/1471-2164-14-836 24279309 PMC4046679

[35] Kitashiba H, Li F, Hirakawa H, Kawanabe T, Zou Z, Hasegawa Y, et al Draft sequences of the radish (*Raphanus sativus* L.) genome. DNA research. 2014;21(5):481–90. 10.1093/dnares/dsu014 24848699 PMC4195494

[36] Mitsui Y, Shimomura M, Komatsu K, Namiki N, Shibata-Hatta M, Imai M, et al The radish genome and comprehensive gene expression profile of tuberous root formation and development. Scientific Reports. 2015;5:10835 10.1038/srep10835 26056784 PMC4650646

[37] Florea L, Song L, Salzberg SL. Thousands of exon skipping events differentiate among splicing patterns in sixteen human tissues. F1000Research. 2013;2:188 10.12688/f1000research.2-188.v2 24555089 PMC3892928

[38] Schulze SK, Kanwar R, Golzenleuchter M, Therneau TM, Beutler AS. SERE: single-parameter quality control and sample comparison for RNA-Seq. BMC Genomics. 2012;13:524 10.1186/1471-2164-13-524 23033915 PMC3534338

[39] Anders S, Huber W. Differential expression analysis for sequence count data. Genome Biology. 2010;11(10):R106 10.1186/gb-2010-11-10-r106 20979621 PMC3218662

[40] Chanter D. Kolmogorov-Smirnov tests. Teaching Statistics. 1990;12(3):90.

[41] Wang M, Zhang X, Liu JH. Deep sequencing-based characterization of transcriptome of trifoliate orange (*Poncirus trifoliata* (L.) Raf.) in response to cold stress. BMC Genomics. 2015;16:555 10.1186/s12864-015-1629-7 26219960 PMC4518522

[42] Kim DH, Sung S. Coordination of the vernalization response through a VIN3 and FLC gene family regulatory network in *Arabidopsis*. The Plant Cell. 2013;25(2):454–69. 10.1105/tpc.112.104760 23417034 PMC3608771

[43] Krogan NJ, Dover J, Wood A, Schneider J, Heidt J, Boateng MA, et al The Paf1 complex is required for histone H3 methylation by COMPASS and Dot1p: linking transcriptional elongation to histone methylation. Molecular Cell. 2003;11(3):721–9. 12667454 10.1016/s1097-2765(03)00091-1

[44] Cao Y, Dai Y, Cui S, Ma L. Histone H2B monoubiquitination in the chromatin of *FLOWERING LOCUS C* regulates flowering time in *Arabidopsis*. The Plant Cell. 2008;20(10):2586–602. 10.1105/tpc.108.062760 18849490 PMC2590739

[45] Betz JL, Chang M, Washburn TM, Porter SE, Mueller CL, Jaehning JA. Phenotypic analysis of Paf1/RNA polymerase II complex mutations reveals connections to cell cycle regulation, protein synthesis, and lipid and nucleic acid metabolism. Molecular Genetics and Genomics. 2002;268(2):272–85. 10.1007/s00438-002-0752-8 12395202

[46] Choi K, Kim J, Hwang HJ, Kim S, Park C, Kim SY, et al The FRIGIDA complex activates transcription of FLC, a strong flowering repressor in Arabidopsis, by recruiting chromatin modification factors. The Plant Cell. 2011;23(1):289–303. 10.1105/tpc.110.075911 21282526 PMC3051252

[47] Margueron R, Reinberg D. The polycomb complex PRC2 and its mark in life. Nature. 2011;469(7330):343–9. 10.1038/nature09784 21248841 PMC3760771

[48] Jiang D, Wang Y, Wang Y, He Y. Repression of *FLOWERING LOCUS C* and *FLOWERING LOCUS T* by the Arabidopsis Polycomb repressive complex 2 components. PloS Cne. 2008;3(10):e3404. 10.1371/journal.pone.0003404 PMC2561057 18852898

[49] Barth C, Jander G. Arabidopsis myrosinases TGG1 and TGG2 have redundant function in glucosinolate breakdown and insect defense. The Plant Journal. 2006;46(4):549–62. 10.1111/j.1365-313X.2006.02716.x 16640593

[50] Su H, Xu K, Liu W. Changes of endogenous hormones during the process of flower bud differentiation of *Welsh Onion*. Acta Horticulturae Sinica. 2007;34(3):671–6.

[51] Wang L, Pang J, Zhu M, Hu J. Studies on the critical period of floral differentiation and changes of endogenous hormone contents in the decapitated seedlings of cucumber. Journal of Zhejiang University. 2004;31(2):202–6.

[52] Kurepin LV, Dahal KP, Savitch LV, Singh J, Bode R, Ivanov AG, et al Role of CBFs as integrators of chloroplast redox, phytochrome and plant hormone signaling during cold acclimation. International Journal of Molecular Sciences. 2013;14(6):12729–63. 10.3390/ijms140612729 23778089 PMC3709810

[53] Knox AK, Li C, Vagujfalvi A, Galiba G, Stockinger EJ, Dubcovsky J. Identification of candidate CBF genes for the frost tolerance locus *Fr-Am2* in *Triticum monococcum*. Plant Molecular Biology. 2008;67(3):257–70. 10.1007/s11103-008-9316-6 18317935

[54] Vagujfalvi A, Aprile A, Miller A, Dubcovsky J, Delugu G, Galiba G, et al The expression of several *Cbf* genes at the *Fr-A2* locus is linked to frost resistance in wheat. Molecular Genetics and Genomics. 2005;274(5):506–14. 10.1007/s00438-005-0047-y 16200412

[55] Fowler S. Arabidopsis transcriptome profiling indicates that multiple regulatory pathways are activated during cold acclimation in addition to the CBF cold response pathway. The Plant Cell. 2002;14(8):1675–90. 10.1105/tpc.003483 12172015 PMC151458

[56] Kim D, Pertea G, Trapnell C, Pimentel H, Kelley R, Salzberg SL. TopHat2: accurate alignment of transcriptomes in the presence of insertions, deletions and gene fusions. Genome Biology. 2013;14(4):R36 10.1186/gb-2013-14-4-r36 23618408 PMC4053844

[57] Trapnell C, Williams BA, Pertea G, Mortazavi A, Kwan G, van Baren MJ, et al Transcript assembly and quantification by RNA-Seq reveals unannotated transcripts and isoform switching during cell differentiation. Nature Biotechnology. 2010;28(5):511–5. 10.1038/nbt.1621 20436464 PMC3146043

[58] Pfaffl MW. A new mathematical model for relative quantification in real-time RT-PCR. Nucleic Acids Research. 2001;29(9):e45 11328886 10.1093/nar/29.9.e45 PMC55695

