# Supplementary figures and images for: Genome-wide transcriptome profiling of radish (Raphanus sativus L.) in response to vernalization
Source: PLoS One. 2017 May 12;12(5):e0177594. doi: 10.1371/journal.pone.0177594 (PMC5428929; doi:10.1371/journal.pone.0177594)

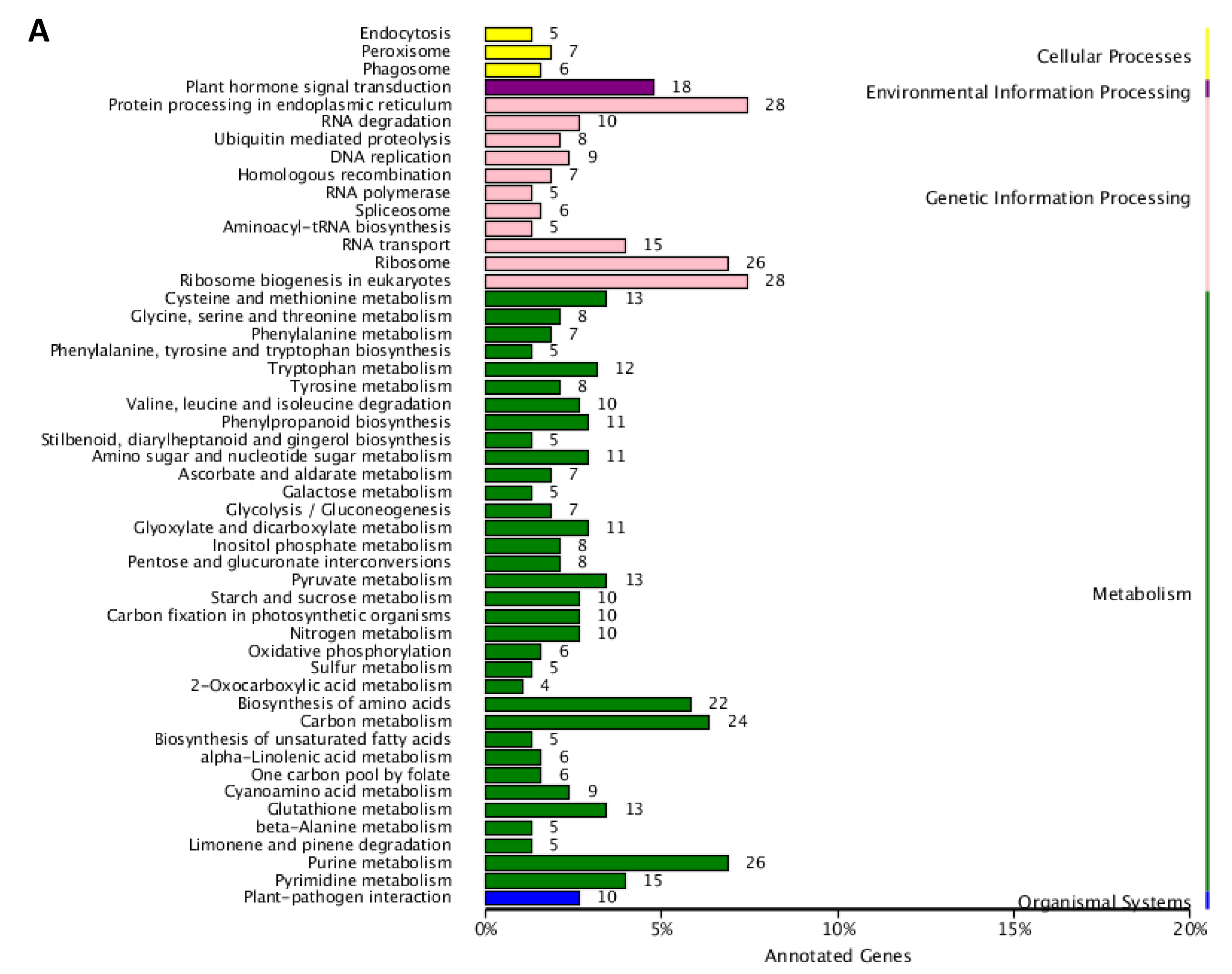

Supplement: S1 Fig — (JPG) [file pone.0177594.s001.jpg]

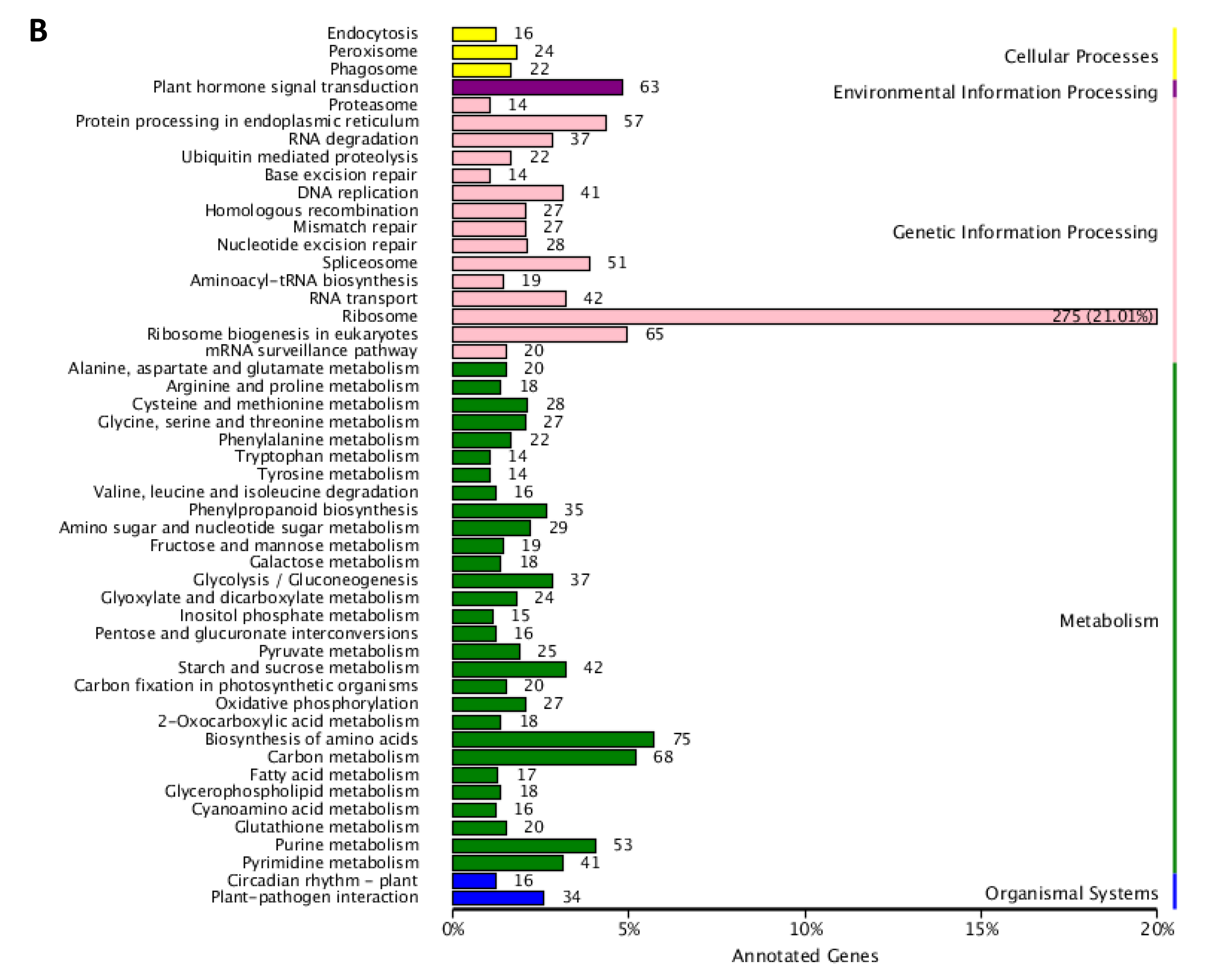

Supplement: S2 Fig — (JPG) [file pone.0177594.s002.jpg]

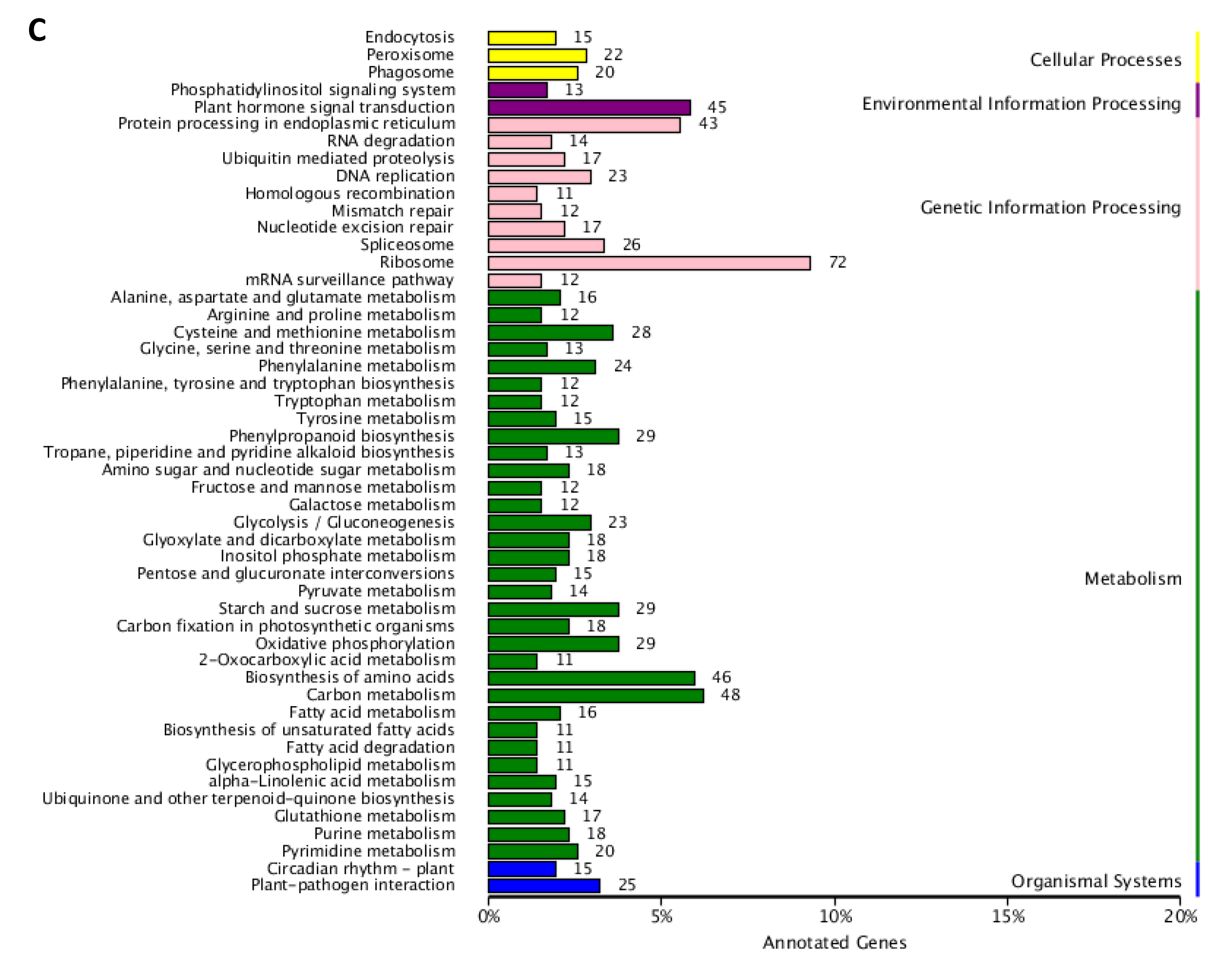

Supplement: S3 Fig — (JPG) [file pone.0177594.s003.jpg]

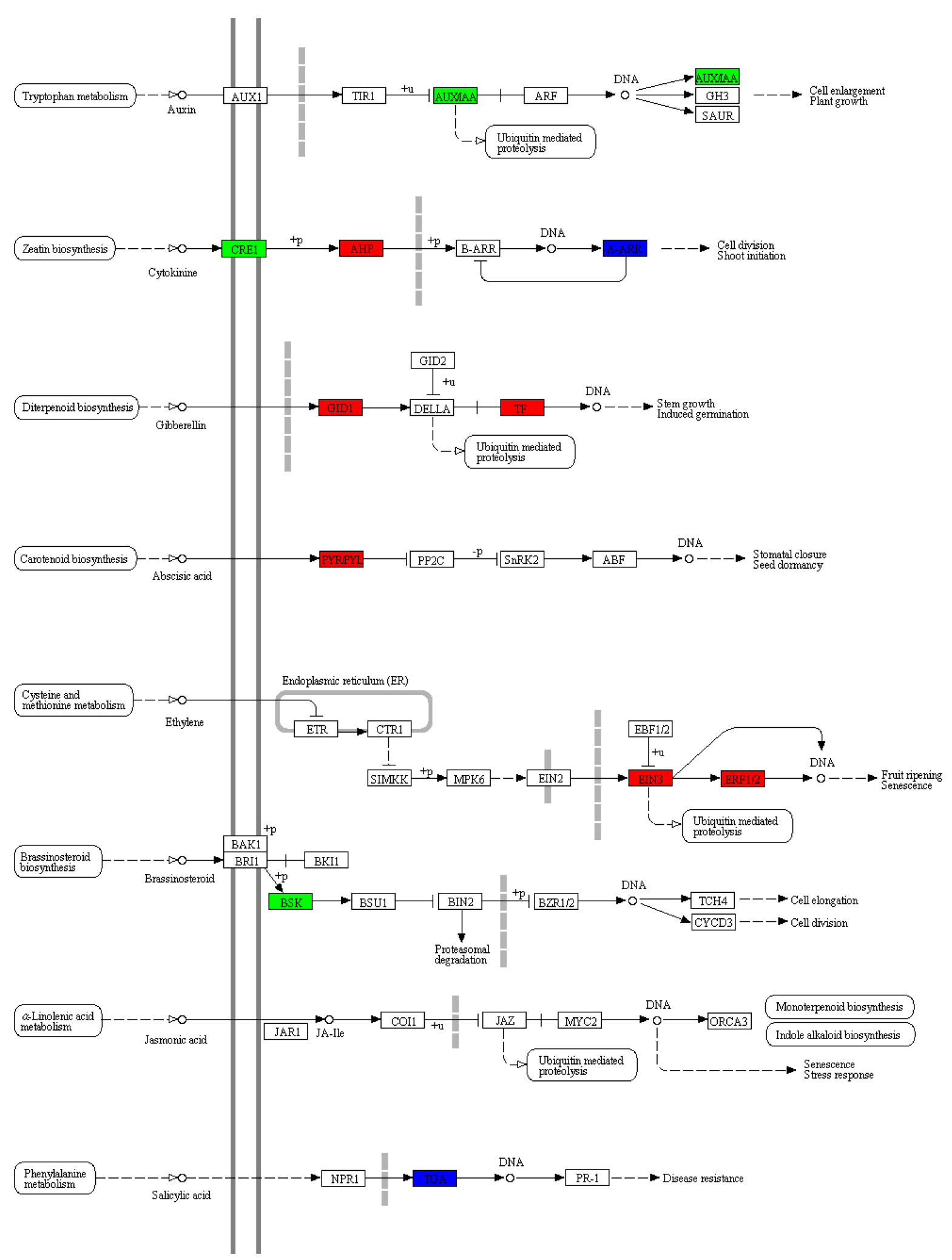

Supplement: S4 Fig — (JPG) [file pone.0177594.s004.jpg]
